# The national distribution of lymphatic filariasis cases in Malawi using patient mapping and geostatistical modelling

**DOI:** 10.1371/journal.pntd.0012056

**Published:** 2024-03-25

**Authors:** Carrie Barrett, John Chiphwanya, Square Mkwanda, Dorothy E. Matipula, Paul Ndhlovu, Limbikani Chaponda, Joseph D. Turner, Emanuele Giorgi, Hannah Betts, Sarah Martindale, Mark J. Taylor, Jonathan M. Read, Louise A. Kelly-Hope

**Affiliations:** 1 Centre for Neglected Tropical Disease, Department of Tropical Disease Biology, Liverpool School of Tropical Medicine, Pembroke Place, Liverpool, United Kingdom; 2 National Lymphatic Filariasis Elimination Programme, Ministry of Health, Lilongwe, Malawi; 3 Lancaster Medical School, South West Drive, Bailrigg, Lancaster, United Kingdom; 4 Department of Livestock and One Health, Institute of Infection, Veterinary and Ecological Sciences, University of Liverpool, Liverpool, United Kingdom; Federal University of Ceará, Fortaleza, Brazil, BRAZIL

## Abstract

**Background:**

In 2020 the World Health Organization (WHO) declared that Malawi had successfully eliminated lymphatic filariasis (LF) as a public health problem. Understanding clinical case distributions at a national and sub-national level is important, so essential care packages can be provided to individuals living with LF symptoms. This study aimed to develop a national database and map of LF clinical cases across Malawi using geostatistical modelling approaches, programme-identified clinical cases, antigenaemia prevalence and climate information.

**Methodology:**

LF clinical cases identified through programme house-to-house surveys across 90 sub-district administrative boundaries (Traditional Authority (TA)) and antigenaemia prevalence from 57 sampled villages in Malawi were used in a two-step geostatistical modelling process to predict LF clinical cases across all TAs of the country. First, we modelled antigenaemia prevalence in relation to climate covariates to predict nationwide antigenaemia prevalence. Second, we modelled clinical cases for unmapped TAs based on our antigenaemia prevalence spatial estimates.

**Principle findings:**

The models estimated 20,938 (95% CrI 18,091 to 24,071) clinical cases in unmapped TAs (70.3%) in addition to the 8,856 (29.7%), programme-identified cases in mapped TAs. In total, the overall national number of LF clinical cases was estimated to be 29,794 (95% CrI 26,957 to 32,927). The antigenaemia prevalence and clinical case mapping and modelling found the highest burden of disease in Chikwawa and Nsanje districts in the Southern Region and Karonga district in the Northern Region of the country.

**Conclusions:**

The models presented in this study have facilitated the development of the first national LF clinical case database and map in Malawi, the first endemic country in sub-Saharan Africa. It highlights the value of using existing LF antigenaemia prevalence and clinical case data together with modelling approaches to produce estimates that may be used for the WHO dossier requirements, to help target limited resources and implement long-term health strategies.

## Author summary

Lymphatic filariasis (LF) is a disfiguring and painful Neglected Tropical Disease, transmitted by mosquitoes, and impairs affected individual’s mental wellbeing, social participation, and ability to work. The two most common clinical manifestations are hydrocoele (scrotal swelling) and lymphoedema (swelling of the limbs). Estimates of LF clinical case numbers are required to provide national and local care needs assessment, and for elimination and surveillance purposes. Clinical case prevalence is currently not readily available or is unknown across many sub-Saharan African countries, however Malawi is unique as the LF Programme has conducted extensive house-to-house patient mapping activities across one third of the country. We used this clinical data in combination with measurements of LF infection prevalence and high-resolution climate information, to develop geostatistical models, which estimate the number of clinical cases in unmapped areas. This led to the development of a national database and map of clinical case estimates that will help the Malawi LF Elimination Programme to optimize limited resources, target morbidity management and disability prevention, and improve quality of life of the people affected by this disabling and disfiguring disease.

## Introduction

In sub-Saharan Africa lymphatic filariasis (LF) is a mosquito-borne disease caused by the parasitic nematode, *Wuchereria bancrofti* [1,2]. LF is targeted for elimination in 27 African countries (77%) by 2030 described in the World Health Organization (WHO) Neglected Tropical Disease (NTD) road map 2021–2030 [3,4]. To achieve validation of elimination of LF as a public health problem granted by the WHO, countries are required to submit and meet WHO dossier requirements [5]. In addition, countries must be implementing post-validation activities for surveillance and integrating morbidity management into existing health systems [5–7]. In 2018, approximately 51 million individuals were estimated to be infected with LF, which has reduced by 74% since the Global Programme to Eliminate LF (GPELF) began [8]. In 2000, clinical case estimates in sub-Saharan Africa ranged from 46 to 51 million, which are now outdated and clinical case estimates are unavailable in many countries [9]. The two most common chronic clinical manifestations of LF are hydrocoele (scrotal swelling) and lymphoedema (swelling of the limbs) that cause pain, profound disfigurement and large financial, social and mental health losses [2,10].

The Malawi LF Elimination Programme has achieved certification of LF elimination as a public health problem in 2020 from the WHO [3,11]. Over the past two decades the programme successfully implemented prevalence mapping, effective anti-filarial mass drug administration (MDA), impact assessments, morbidity management and disability prevention (MMDP) and operational research activities as outlined in Chiphwanya et al. [11]. The widespread endemic nature of LF across the country became evident in the early 2000s when LF antigenaemia prevalence surveys were conducted in villages using antigen-based immunochromatographic rapid tests (ICTs), although this data was geographically sparse in comparison to large district areas [12–14]. More recently, attention has focussed on obtaining better estimates of clinical burden, and the programme conducted a series of large-scale house-to-house mapping activities, across 23 districts in 90 sub-district administrative areas known as Traditional Authorities (TAs) between 2014–2021. The extensive clinical case mapping covered an area of over 33,000km^2^ populated by 5.6 million people, representing approximately 35% of the geographical area of Malawi, identifying 8,856 clinical cases: male hydrocoele = 6,333 (71.5%; average age 50.5); male lymphoedema = 854 (9.6%; average age 54.4; female lymphoedema = 1,585 (17.9%; average age 50.5); male both = 84 (1.0%; average age 58.8) [11].

In Malawi, estimating the number of hydrocoele and lymphoedema cases was important to allow for the planning and provision of services that are available within the primary care system in all areas with known affected people, in line with the WHO essential package of care recommendations which include: hydrocoele surgeries; treatment for episodes of adenolymphangitis (ADL) through antibiotic treatment and symptomatic management; management of lymphoedema (trained health workers able to provide and teach patients self-care measures of hygiene, skin and wound-care, elevation, and exercise) [15]. In addition, short term studies have shown the inclusion of diaphragmatic deep-breathing exercises, lymphatic massage, and dietary changes to improve lymphoedema status and significantly reduce frequency and duration of ADLs [16–18]. The case estimates also helped to direct actions and provide documentation for the WHO dossier on (1) the number of hydrocoele and lymphoedema case estimates in all endemic implementation units (IUs); (2) assess the availability and quality of available funding and resources to (3) provide full geographical coverage of essential package of care within all endemic IUs [5].

In many endemic countries clinical case estimates are lacking, which is likely due to the significant time, human, and financial resources required [10]. Only one country in Asia has developed a national database and map of LF cases, and also found that disease prevalence was positively correlated with antigenaemia prevalence (prior to MDA) [6], which supports findings from historical studies [19,20]. The close relationship between disease and antigenaemia prevalence suggests that these data may be used together to model and predict clinical cases and prevalence rates in unmapped TAs. The use of multiple types of prevalence data has proven useful when data is sparse and resources limited, as shown in other disease mapping activities [21]. In addition, the use of climate covariates such as temperature, rainfall, humidity, may also help model predictions as found in antigenaemia and microfilaria prevalence studies as they impact the abundance of mosquito vectors and LF transmission rates, therefore may be useful in understanding disease distributions and risk [22–25].

To support the Malawi LF Elimination Programme with obtaining LF case estimates across all endemic areas of the country, this study aimed to develop a national map of LF clinical cases, using a geostatistical modelling approach from a combination of clinical case data, antigenaemia prevalence and climate information. A two-step geostatistical analysis was conducted. First, we modelled antigenaemia prevalence (prior to MDA) in relation to climate covariates to predict national antigenaemia prevalence. Second, we modelled clinical cases for unmapped TAs based on these antigenaemia prevalence spatial estimates.

## Methods

### Ethics statement

Ethical approval was obtained from the Research Ethics Committee at Liverpool School of Tropical Medicine, UK (protocol number 12.22) and the National Health Sciences Research Committee, Ministry of Health, Malawi (protocol number 1260).

### Data sources

#### LF clinical case data

LF clinical case data was collected across 90 TA areas approximately 35% of Malawi, between 2014 and 2017. National health surveillance assistants, trained to identify and report individual clinical cases (lymphoedema and hydrocoele) using an innovative phone reporting tool [11,26,27]. Data were reported by health facility catchments, which were generally aligned with TA administrative boundaries defined by Humanitarian Data Exchange [28]. Two instances where TA and health facility catchment boundaries were not continuous were resolved by merging TA boundaries for analysis purposes.

#### LF antigenaemia data

The LF antigenaemia prevalence data was collected from three surveys conducted in selected 57 villages across all districts in Malawi between 2000 and 2003, prior to initiation of MDA [12,13,29]. The ICT diagnostic tool was used to detect the presence of *W*. *bancrofti*-specific circulating filarial antigen in whole-blood samples. Details on the surveys’ methodology are available in the original studies [12,13,29]. 21 TAs with available antigenaemia data and LF clinical case data was summarised in S2 Table.

### Statistical analysis

#### Analytical overview

Exploratory analysis identified antigenaemia as a predictor of clinical case prevalence in TAs where both observations were available, see S1 File. However, antigenaemia data was not available for all areas of Malawi (only 57 villages). Step 1: A geostatistical model was fitted to available antigenaemia prevalence data which incorporated climate covariates to predict and map antigenaemia prevalence in areas with and without available antigenaemia prevalence data. Step 2: A second geostatistical model using the predicted antigenaemia prevalence from step 1 was used to predict LF clinical case estimates.

#### Predicting antigenaemia prevalence–geostatistical model 1

To predict antigenaemia prevalence in areas with and without data available data, we used available antigenaemia data and climate covariates identified from the literature and based on the assumption of their impact on mosquito vector abundance and LF transmission rates [24,25,30–32]. We fitted a geostatistical model of antigenaemia prevalence, *P*(*x*), where *x*_*i*_ is the geolocation of village *i*. Available antigenaemia prevalence was determined by the number of positives, *Y*, divided by the number of individuals tested, *N*, following a binomial distribution. Climate covariates: average annual temperature (°C); average annual humidity (kPa); elevation (m); and average annual rainfall (mm) between 1970–2000 were obtained from World Clim, a database for 1km spatial resolution climate surfaces for global land areas. [33]. A Principle Component Analysis (PCA) was performed on the climate covariates to mitigate for collinearity ─ the non-independence of predictor covariates [34]. The first component of the PCA of the chosen climate covariates for each village location, *d*(*x*), was the explanatory variable. To explain the spatial variation in antigenaemia prevalence we include an unobserved stochastic process, *S*(*x*), to represent the variation in *P*(*x*) that is not explained by *d*(*x*). Finally, random variation *U* assuming a Normal Distribution was included in the model, which gave the following step one geostatistical equation:

$$log\left(\frac{P(x_{i})}{1-P(x_{i})}\right)=d(x_{i})+S(x_{i})+U_{i}$$ (Eq 1)

#### Predicting LF clinical cases–geostatistical model 2


$$M(x_{j}^{*})=a{\left(x_{j}^{*}\right)}^{\tau }\beta +log(Pop(x_{j}^{*}))+S(x_{j}^{*})+U_{j}$$ (Eq 2)

To predict LF clinical cases in the TA with no data (i.e., unmapped), we fitted a second geostatistical model of LF clinical cases *M*(x*) using available clinical case data, where $x_{j}^{*}$ is the geolocation for each TA, *j*, assuming a Poisson distribution. Note x* in geostatistical model 2 has a different spatial scale to *x* in geostatistical model 1. The explanatory variable, *a*(x*), predicted antigenaemia prevalence, from geostatistical model 1 was fitted to each centroid geolocation of TA, x* with available clinical case data, including an offset term of the population size (*Pop*) for each TA, taken from 2018 census mapping [35]. An unobserved stochastic process, *S*(x*) was included in the model to represent the variation in *M*(x*) that is not explained by *a*(x*). Finally, random variation *U* assuming a Normal distribution was included in the model to give the step two geostatistical equation.

All geostatistical analyses were performed in R programming software using the PrevMap R package [36,37]. To assess the goodness of fit of the geostatistical model 2 predictions, the mean predicted LF clinical cases were compared to programme-collected clinical cases numbers within TAs observations [11]. Within S2 File, the goodness of fit of the geostatistical model 1 predictions were assessed by comparison against antigenaemia prevalence.

### Mapping

Three maps were produced, the first showing predicted antigenaemia prevalence (%) from the geostatistical model 1 using QGIS software 3.22.5 [38] on 4.5km (longitude) x 4.6km (latitude) scale. The second map showing LF clinical cases and the third showing LF clinical case prevalence per 100,000 population [35] for TA areas [28] from geostatistical model 2 predictions and available programme-collected data.

### National estimates

National and TA boundary LF clinical case estimates and prevalence (case per 100,000 population) were summarised from geostatistical model predictions for the 320 unmapped TA areas. S1 Table summarises this for each TA boundary in Malawi. The 95% credible intervals for national LF clinical case estimates were calculated from geostatistical model predictions and summarised for predicted case numbers. National sex-specific hydrocoele and lymphoedema clinical cases were estimated by partitioning the national estimated LF clinical cases by programme-identified observed proportions.

## Results

### Antigenaemia prevalence mapping–geostatistical model 1

**Fig 1:**
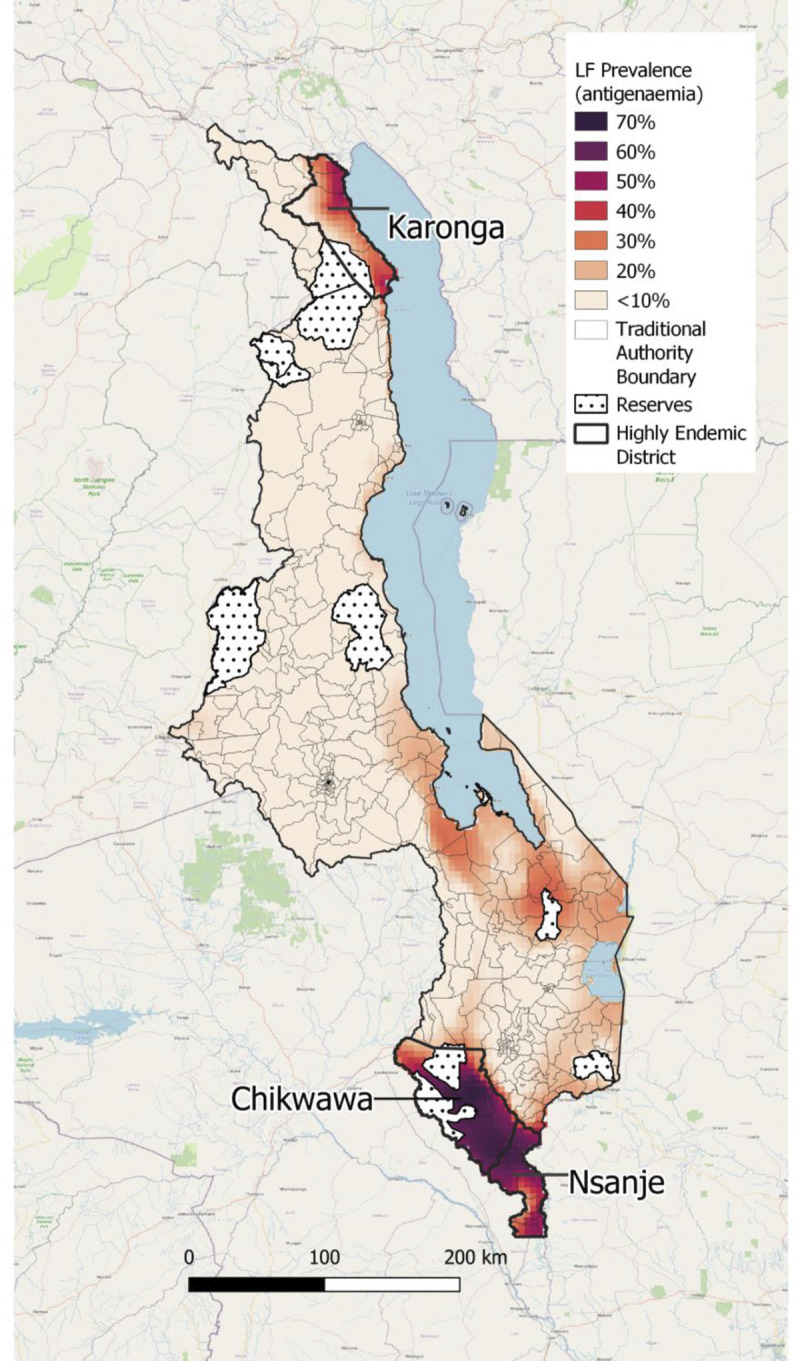
LF prevalence (antigenaemia) map from step one geostatistical analysis. Note: Maps were produced in QGIS mapping software (https://qgis.org) using the base layer from OpenStreetMap (https://www.openstreetmap.org/), and country administrative boundaries available from the Humanitarian Data Exchange [28].

We found a positive association between antigenaemia prevalence and clinical case prevalence within TAs, see S1 File. The antigenaemia prevalence predicted from the first geostatistical model using climate covariates was mapped across Malawi and is presented in Fig 1. LF antigenaemia prevalence varied between 0.5% and 69.3% across the country on a spatial scale of 4.5km x 4.6km cells. For the majority of the country the prevalence was low (0.9% ─ 15.7%), however the prevalence was high (1.7% ─ 50.5%) in the northern district Karonga, districts along the southern shore of Lake Malawi (Salima, Dedza, Ntcheu, Mangochi, Balaka and Machinga) (1.4% ─ 33.0%), and highest in the southern districts of Chikwawa and Nsanje (16.4% ─ 69.3%).

### Clinical case mapping–geostatistical model 2

**Fig 2:**
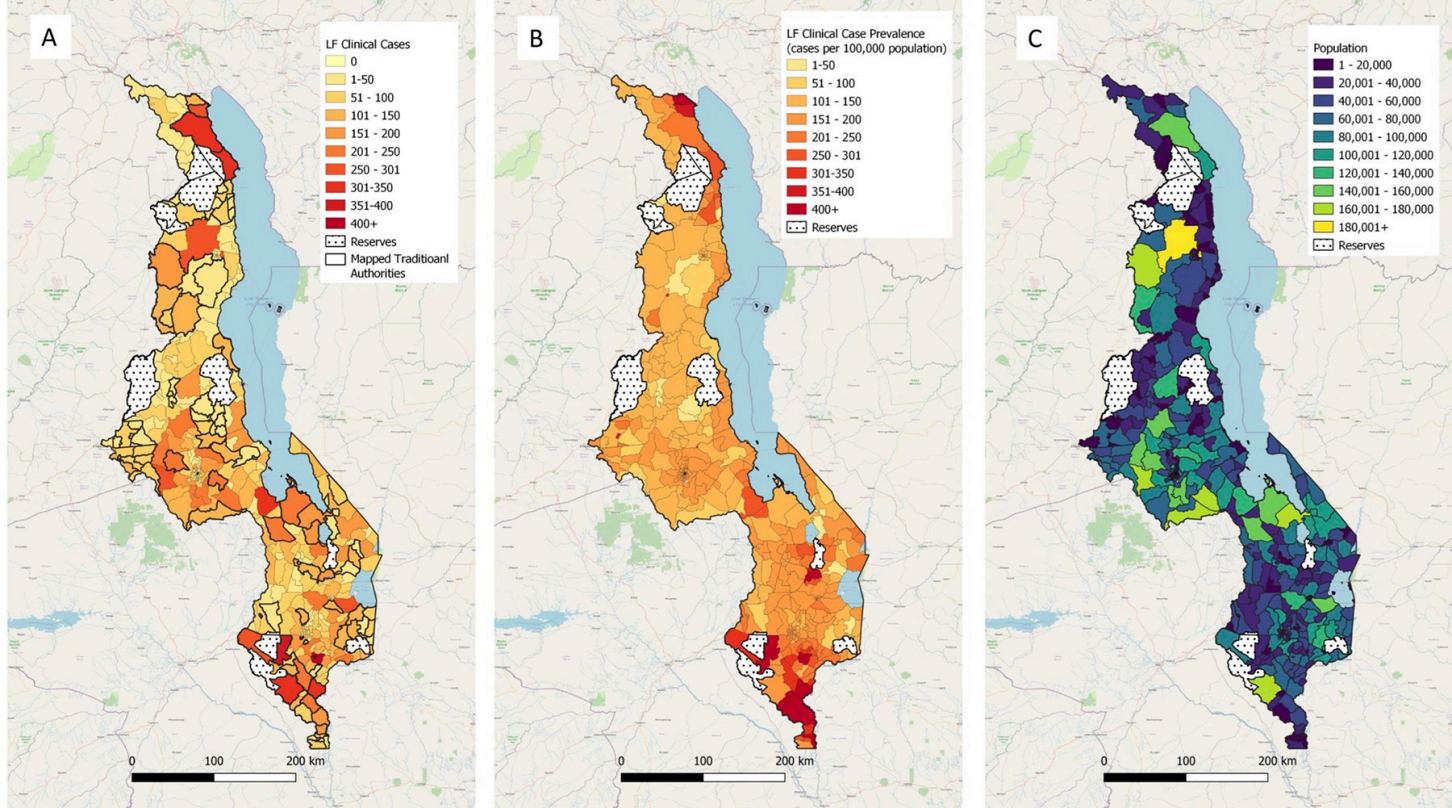
National LF clinical case (A) numbers and (B) prevalence (cases per 100,000 population) and (C) population size taken from Census Report in 2018 [35] summarised at Traditional Authority level.

The number of predicted LF clinical cases for each TA across Malawi from the second geostatistical model was presented in Fig 2A. The predicted LF clinical case prevalence per 100,000 population for each TA is presented in Fig 2B. The number of cases within each TA were summarised in S1 Table.

The number of LF clinical cases varied between TAs, particularly within the middle region of the country, in areas along the southern shore of Lake Malawi and programme-collected TA data, however the prevalence of cases is more consistent amongst predicted TAs in the middle region of the country, suggesting differing population size does impact the number of cases found within each TA. Two TAs with the highest number of LF clinical cases in the middle region of the country were in Lilongwe district, called TA Mazengera and TA Kabudula. Similar to the antigenaemia prevalence, LF clinical cases and case prevalence were high in the northern Karonga district, areas along the southern shore of Lake Malawi, and highest in the southern districts of Chikwawa and Nsanje.

Note: Maps were produced in QGIS mapping software (https://qgis.org) using the base layer from OpenStreetMap (https://www.openstreetmap.org/), and country administrative boundaries available from the Humanitarian Data Exchange [28].

### Assessing the goodness of fit

**Fig 3:**
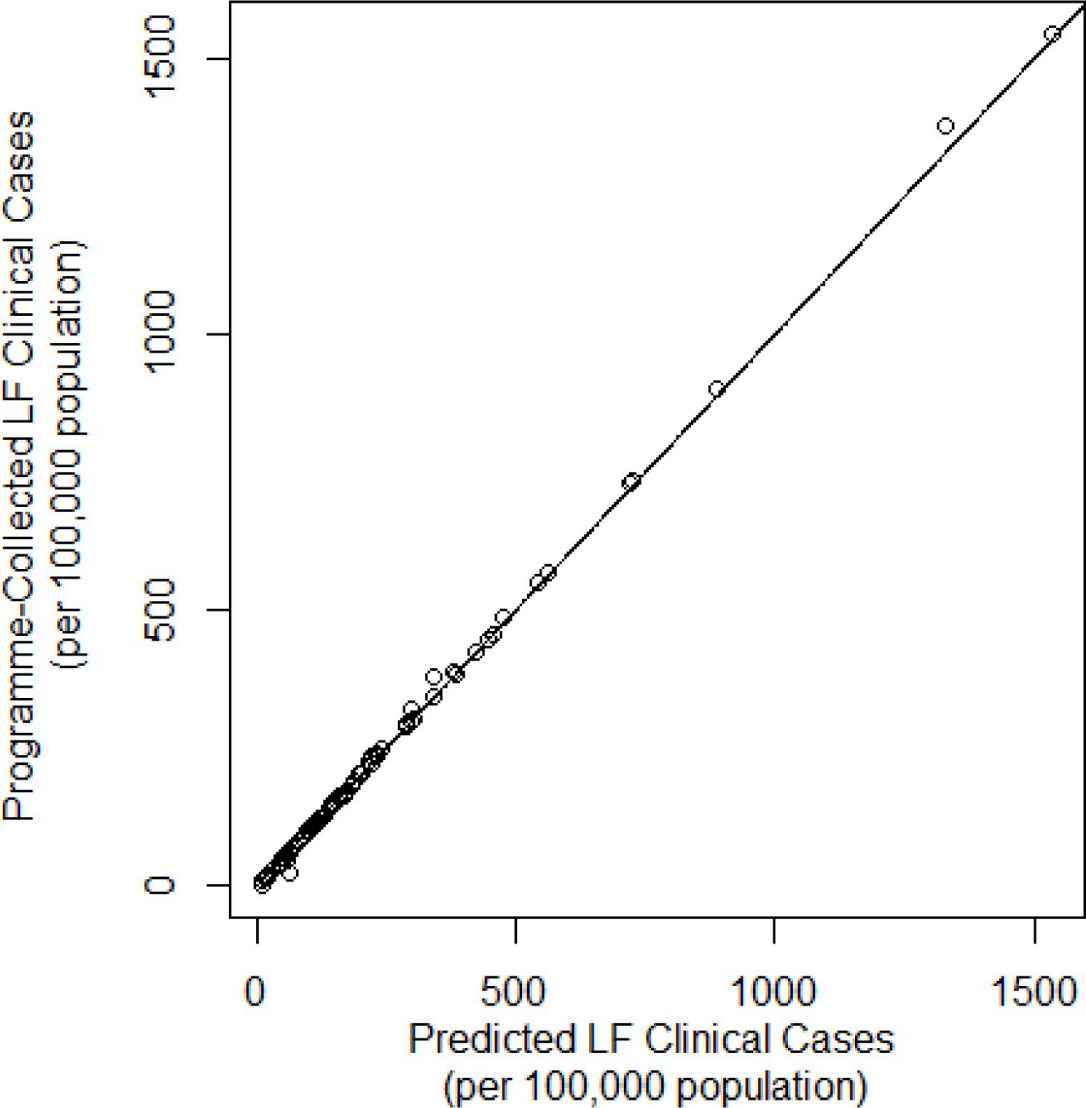
Predicted clinical case prevalence from geostatistical analysis compared against programme identified LF clinical case prevalence per 100,000 population.

To assess the goodness of fit of the geostatistical model in step 2, the predicted prevalence LF clinical cases was compared to the number of programme identified clinical case prevalence per 100,000 population shown in Fig 3. Many TA observations that fell on, or very close to, the line, suggesting that the geostatistical model has a good predictive ability.

### National LF clinical cases

From geostatistical modelling, the overall estimated number of LF clinical cases was 29,794 calculated with 95% credible intervals (CrI) 26,957 to 32,927 across all endemic districts in Malawi. The geostatistical analysis identified a further estimated 20,938 (95% CrI 18,091 to 24,071) cases in the 320 unmapped TA areas (70.3%), in addition to the 8,856 cases identified by the programme house-to-house surveys (29.7%), see Table 1. We estimated that there were 21,306 (71.5%) male hydrocoele cases, 2,873 (9.6%) male lymphoedema, 5,332 (17.9%) female lymphoedema and 283 (1.0%) male both.

**Table 1:** LF clinical cases across mapped and unmapped Traditional Authority (TA) areas in Malawi.

	Clinical cases	95% Credible Interval	Total Population*	Clinical Case Prevalence (cases per 100,000 population)
Mapped TA Areas	8,856	NA	5,613,230	158
Unmapped TA Areas	20,938	18,091–24,071	11,950,519	175
All TA Areas	29,794	26,957–32,927	17,563,749	170

* Population size taken from Census Report in 2018 [35].

## Discussion

This study makes Malawi the first LF endemic African country to produce a national level database of LF clinical cases, estimating 29,794 (26,957–32,927) from the 8,856 (29.7%) programme-identified cases and 20,938 (18,091–24,071) cases predicted from geostatistical analysis. As well as producing a set of risk maps of LF clinical case and antigenaemia prevalence estimates. The highest numbers of LF clinical cases and antigenaemia prevalence from geostatistical model predictions and programme data were observed in Chikwawa and Nsanje districts in the Southern Region and Karonga district in the Northern Region of the country. The results presented in this study provide the Malawi LF programme and health system with an informative understanding of the clinical case distributions across local regions, allowing them to target resources for MMDP in identified high risk TA areas, where cases were predicted as high as 524 (Thyolo district, TA Nchilamwela).

The maps produced in this study demonstrate the widespread distribution of LF clinical cases and prevalence across Malawi. The detailed antigenaemia prevalence map was produced from data collected between 2000–2003, prior to MDA, using climate information from 1970–2000. Following implementation of MDA initiated in 2008, antigenaemia prevalence has decreased [39]. The map shows the highest LF case prevalence occurred in the northern region in Karonga district and southern region, Nsanje and Chikwawa districts, which may be due to these areas having optimum climate conditions that drive transmission and abundance of mosquito vectors. Within these southern regions of Malawi, Nsanje and Chikwawa districts, predominantely *Anopheles funestus*, as well as *A*. *arabiensis* and *A*. *gambiae* have been categorised as main LF vectors [11,40]. Along Lake Malawi shore, antigenaemia prevalence was observed to be higher compared to in-land areas, thus likely due to mosquito breeding sites driving *W*. *bancrofti* infection as well as human populations inhabiting areas close to water bodies [41].

From LF clinical case predictions and programmatic data presented in this study, the highest number of cases occurred within districts with highest antigenaemia prevalence, Chikwawa, Nsanje and Karonga. However, middle regions of Malawi showed a higher prevalence of cases where antigenaemia prevalence was found to be low, suggesting that population may play an important role in defining risk areas for clinical cases. In Bangladesh similar gender ratios and age distributions have been observed [6]. Higher proportions of LF clinical case prevalence been found to be associated with people living in rural areas, poverty, and poor sanitation [42]. Similar comparisons can be drawn to highly endemic districts in Malawi, Chikwawa and Nsanje, although more research is needed to identify risk factors for LF cases within this context. S1 Table describes the case predictions made from this study analysis predictions and programme-identified cases for each TA boundary in Malawi. Our findings suggest that historic antigenaemia prevalence may be a good predictor for highly endemic regions, but more research is needed from other countries and ecological zones to solidify this relationship [6,19,20].

Our geostatistical approach offers an alternative to national patient searching. This approach may be an improvement over other methods, i.e. community drug distributor estimations of cases during MDA programmes, which potentially underestimate clinical cases [43,44]. In the absence of expertise and resources for more complex geostatistical modelling, we advocate that antigenaemia prevalence data could be used to estimate LF clinical case distributions. Other country elimination programmes may refer to the S1 File, which features the relationship between antigenemia prevalence and LF clinical cases, and to use this as a guide to estimate clinical cases within their implementation unit boundary if antigenemia prevalence is available. Geostatistical modelling has shown to substantially outperform current WHO guidelines to collect case estimates, in studies of other NTDs, including soil-transmitted helminth infections in Kenya, Sierra Leone, and Zimbabwe [45]. Geostatistical modelling approaches can offer improved precision of cases estimates for a reduced field-sampling effort, particularly useful for NTDs where resources are often limited. More work utilising geostatistical approaches to estimate and map clinical cases in sub-Saharan Africa would be beneficial as current estimates are limited and many countries have antigenaemia data that could be used [4].

For men with hydrocoeles, we estimate thousands of men living with symptoms are likely to require surgery in the next decade. Assessing the capacity of hospitals, the infrastructure, human and financial resources required for these surgeries is important to determine whether they can be provided routinely through the health system. A hospital facility assessment to determine the readiness and quality of hydrocelectomy services has been conduced in Malawi in 2019 [46]. To reduce the backlog of cases, hydrocoele campaigns were conduced periodically from 2008 completing more than 1500 surgeries in high burden districts, Karonga, Chikwawa and Nsanje [11]. In Malawi, hydrocoele surgeries were found to have significant improvements in men’s quality of life, as well as life-time economic benefits to the individual, his family, and his community which greatly outweigh the low cost of surgery (estimated at US$68 during campaigns) [47,48]. However, since 2015 when the LF programme hydrocoele campaigns have ceased, the capacity of hospital’s to address the backlog of hydrocoeles remains a challenge in TA’s where LF clinical cases are high.

For people with lymphoedema, life-long home-based MMDP strategies is required to manage their and ADLs, to prevent and hinder progression of their symptoms [15]. Studies have shown that lymphoedema predominately affects women [26,49–51], although the reasons for this are not well understood [52]. The home-based care recommended by WHO includes daily hygiene, skin care, limb exercise and elevation [15], with recent research showing the benefits of additional exercises including lymphatic massage, deep diaphragm breathing techniques, skin mobilization, seated and standing exercises [16–18]. Most mild stages of lymphoedema will manage symptoms with home-based care activities, however the more severe stages require more specialist care [50]. Adopting a holistic approach which addresses the physical, psychological and social implications of lymphoedema is required due to the chronic, stigmatizing nature of this condition [3].

In Malawi, lymphatic management training has been provided to all health surveillance assistants and two staff 259 health centres. More than 4000 community health workers have been trained in lymphoedema management and to provide further training in the lymphoedema home-based care to all persons affected identified within their catchment area. All health centres across the country provide free of charge services during ADL episodes, such as antibiotics or pain relief [11]. The findings from this study show the wide spread distribution LF clinical cases across the country, that will all require lymphoedema management training to hinder the progression of their disease and reduce further disability. The majority of LF clinical cases in Malawi are located within rural communities, where access to healthcare or trained healthcare staff remains a challenge, as well as equitable access to quality healthcare services for women [53]. Additional challenges include addressing the psychosocial consequences of lymphoedema [51].

As many countries move into the LF elimination phase, strategies to focus on alleviating suffering of affected individuals through morbidity management is critical. The Malawi LF programme is in the process of planning and continuing the scale-up of a home-based enhanced self-care in all endemic districts, however more funding is required to continue this vital work. The informative LF case numbers presented in this study will allow the programme to identity where they need to verify the numbers and plan to allocate resources, including training of health staff and health surveillance assistants. This will help the integration of the essential packages of care into health systems for increased sustainability. In addition, it is a WHO post-elimination requirement to estimate LF case numbers at the implementation unit level, as well as conducting post-elimination surveillance [5].

### Limitations and considerations

There are several limitations to this study; we will discuss those related to study analysis first. As there was limited data on antigenaemia and LF clinical case mapping, we did not conduct a validation of our geostatistical prediction. This lack of validation means we could not test the reliability of our predictions, therefore more research needs to be done to validate some of the predicted areas through ground truthing field surveys. Additionally, estimates for sex specific hydrocoele and lymphoedema cases were based on the assumption that there were no differences in gender ratios in different TA areas of Malawi and that the programme collected data distributions of hydrocoele and lymphoedema were the same for our model predictions.

Limitations from a public health perspective include that most endemic country programmes have limited access to geostatistical modelers, which restrict the scale up of such work, however a pool of key experts may be convened and work with national programmes to obtain better estimates. Finally, the generalizability in this study is limited to countries with available clinical case data across endemic regions.

## Conclusion

Malawi is the first LF endemic African country to produce a national level database and a set of risk maps of LF clinical case and antigenaemia prevalence estimates. This has been achieved by the extensive detailed patient mapping conducted by the Malawi LF programme, which was used in combination with LF prevalence data, climate information and geostatistical analytical methods. This study highlights the value of combining different data in resource limited settings to help to save time, human and financial resources. Additionally, these predictions equip the Malawi national programme and Ministry of Health with information to help assess the readiness and quality of services needed to act on and deliver targeted care to the people who need it most. As well as providing lessons for many of country programmes close to eliminating LF as a public health problem.

## Supporting information

S1 STROBE Checklist Strobe statement. (DOC)

S1 Table Predicted LF clinical cases and prevalence for Traditional Authority Boundary Administrative Units in Malawi from predictions from geostatistical analysis or programme-collected data. (XLSX)

S2 Table Traditional Authorities with available antigenaemia prevalence data and LF clinical cases data in Malawi. (DOCX)

S1 File Additional Methods and Results for Exploratory Analysis: Antigenaemia and Clinical Case Prevalence in Malawi. (DOCX)

S2 File Assessing goodness of fit of geostatistical model 1. (DOCX)
