# Supplementary material for: The national distribution of lymphatic filariasis cases in Malawi using patient mapping and geostatistical modelling
Source: PLoS Negl Trop Dis. 2024 Mar 25;18(3):e0012056. doi: 10.1371/journal.pntd.0012056 (PMC11018277; doi:10.1371/journal.pntd.0012056)
Supplement: S1 STROBE Checklist — (DOC) [file pntd.0012056.s001.doc]

STROBE Statement—Checklist of items that should be included in reports of ***cross-sectional studies***

|  | Item No | Recommendation |
| --- | --- | --- |
| **Title and abstract** | 1 | (*a*) Indicate the study’s design with a commonly used term in the title or the abstract **Abstract** |
|  |  | (*b*) Provide in the abstract an informative and balanced summary of what was done and what was found **Abstract** |
| Introduction | | |
| Background/rationale | 2 | Explain the scientific background and rationale for the investigation being reported **Introduction, paragraphs 4, 5** |
| Objectives | 3 | State specific objectives, including any prespecified hypotheses  **Introduction paragraph 5** |
| Methods | | |
| Study design | 4 | Present key elements of study design early in the paper **Methods section – Statistical overview, Analytical overview** |
| Setting | 5 | Describe the setting, locations, and relevant dates, including periods of recruitment, exposure, follow-up, and data collection **Methods – Data Sources** |
| Participants | 6 | (*a*) Give the eligibility criteria, and the sources and methods of selection of participants **Methods section – Data sources** |
| Variables | 7 | Clearly define all outcomes, exposures, predictors, potential confounders, and effect modifiers. Give diagnostic criteria, if applicable **Methods – Predicting antigenemia prevalence – geostatistical model 1** |
| Data sources/ measurement | 8* | For each variable of interest, give sources of data and details of methods of assessment (measurement). Describe comparability of assessment methods if there is more than one group  **Methods section – Data sources, Statistical Analysis, Predicting antigenemia prevalence – geostatistical model 1** |
| Bias | 9 | Describe any efforts to address potential sources of bias **The purpose of the study was to address the geographic bias in LF clinical cases in Malawi. As we were reliant on previously collected data with limited covariate information we were not able to fully address any bias in the antigenemia data collected from field studies that we used in our analysis. Methods section – Statistical Analysis** |
| Study size | 10 | Explain how the study size was arrived at  **Not applicable – this is a secondary data analysis** |
| Quantitative variables | 11 | Explain how quantitative variables were handled in the analyses. If applicable, describe which groupings were chosen and why **Methods section – Statistical Analysis** |
| Statistical methods | 12 | (*a*) Describe all statistical methods, including those used to control for confounding **Methods section – Statistical Analysis** |
|  |  | (*b*) Describe any methods used to examine subgroups and interactions **Methods section – Statistical Analysis, Supplementary File 1** |
|  |  | (*c*) Explain how missing data were addressed **Methods section – Statistical Analysis** |
|  |  | (*d*) If applicable, describe analytical methods taking account of sampling strategy **Methods section – Statistical Analysis** |
|  |  | (*e*) Describe any sensitivity analyses **Not applicable** |
| Results | | |
| Participants | 13* | (a) Report numbers of individuals at each stage of study—eg numbers potentially eligible, examined for eligibility, confirmed eligible, included in the study, completing follow-up, and analysed **Results section - Clinical case mapping – geostatistical model 2, National LF clinical cases, Table S1** |
|  |  | (b) Give reasons for non-participation at each stage **Not applicable** |
|  |  | (c) Consider use of a flow diagram **Not applicable** |
| Descriptive data | 14* | (a) Give characteristics of study participants (eg demographic, clinical, social) and information on exposures and potential confounders **Results section - Clinical case mapping – geostatistical model 2, National LF clinical cases, Table S1** |
|  |  | (b) Indicate number of participants with missing data for each variable of interest **Not applicable** |
| Outcome data | 15* | Report numbers of outcome events or summary measures **Results section - Clinical case mapping – geostatistical model 2, National LF clinical cases, Table S1** |
| Main results | 16 | (*a*) Give unadjusted estimates and, if applicable, confounder-adjusted estimates and their precision (eg, 95% confidence interval). Make clear which confounders were adjusted for and why they were included **Results section - National LF clinical cases** |
|  |  | (*b*) Report category boundaries when continuous variables were categorized **Results section - Antigenemia prevalence mapping – geostatistical model 1** |
|  |  | (*c*) If relevant, consider translating estimates of relative risk into absolute risk for a meaningful time period **Not applicable** |
| Other analyses | 17 | Report other analyses done—eg analyses of subgroups and interactions, and sensitivity analyses **Not applicable** |
| Discussion | | |
| Key results | 18 | Summarise key results with reference to study objectives **Discussion, paragraph 3** |
| Limitations | 19 | Discuss limitations of the study, taking into account sources of potential bias or imprecision. Discuss both direction and magnitude of any potential bias **Discussion, Limitations and considerations** |
| Interpretation | 20 | Give a cautious overall interpretation of results considering objectives, limitations, multiplicity of analyses, results from similar studies, and other relevant evidence **Discussion, paragraphs 2-5, Limitations and considerations** |
| Generalisability | 21 | Discuss the generalisability (external validity) of the study results **Discussion paragraphs 2, 4-5** |
| Other information | | |
| Funding | 22 | Give the source of funding and the role of the funders for the present study and, if applicable, for the original study on which the present article is based **Included in the** **funding information section on online submission** |

*Give information separately for exposed and unexposed groups.

**Note:** An Explanation and Elaboration article discusses each checklist item and gives methodological background and published examples of transparent reporting. The STROBE checklist is best used in conjunction with this article (freely available on the Web sites of PLoS Medicine at http://www.plosmedicine.org/, Annals of Internal Medicine at http://www.annals.org/, and Epidemiology at http://www.epidem.com/). Information on the STROBE Initiative is available at www.strobe-statement.org.
