# Supplementary material for: The national distribution of lymphatic filariasis cases in Malawi using patient mapping and geostatistical modelling
Source: PLoS Negl Trop Dis. 2024 Mar 25;18(3):e0012056. doi: 10.1371/journal.pntd.0012056 (PMC11018277; doi:10.1371/journal.pntd.0012056)
Supplement: S2 Table — (DOCX) [file pntd.0012056.s003.docx]

| **Traditional Authority** | **District** | **No. Examined** | **No. Positive** | **Antigenaemia**  **Prevalence** | **No. of Hydrocele Cases** | **No. of Total LF Clinical Cases** | **Population** | **Male Population** | **Prevalence (All Cases)** | **Hydrocele Prevalence** |
| --- | --- | --- | --- | --- | --- | --- | --- | --- | --- | --- |
| TA Kapeni | Blantyre | 77 | 5 | 0.06 | 53 | 123 | 103742 | 50747 | 0.0012 | 0.0010 |
| TA Chapananga | Chikwawa | 128 | 76 | 0.59 | 229 | 303 | 88641 | 43262 | 0.0034 | 0.0053 |
| TA Kasisi | Chikwawa | 116 | 79 | 0.68 | 225 | 359 | 62323 | 30628 | 0.0058 | 0.0073 |
| TA Makhwira | Chikwawa | 147 | 63 | 0.43 | 180 | 243 | 79933 | 38629 | 0.0030 | 0.0047 |
| TA Maseya | Chikwawa | 315 | 193 | 0.61 | 48 | 69 | 37793 | 18614 | 0.0018 | 0.0026 |
| TA Kilupula | Karonga | 191 | 88 | 0.46 | 166 | 302 | 78424 | 37721 | 0.0039 | 0.0044 |
| TA Kyungu,  Karonga Town | Karonga | 251 | 106 | 0.42 | 254 | 335 | 151281 | 73348 | 0.0022 | 0.0035 |
| TA Mwakaboko | Karonga | 102 | 59 | 0.58 | 83 | 137 | 24889 | 11974 | 0.0055 | 0.0069 |
| TA Mwirang'ombe,  TA Wasambo | Karonga | 101 | 42 | 0.42 | 250 | 318 | 110434 | 53154 | 0.0029 | 0.0047 |
| TA Kaomba | Kasungu | 65 | 0 | 0 | 6 | 10 | 51295 | 25282 | 0.0002 | 0.0002 |
| TA Mponda | Mangochi | 90 | 12 | 0.13 | 138 | 188 | 167313 | 80204 | 0.0011 | 0.0017 |
| TA Mavwere | Mchinji | 98 | 4 | 0.04 | 33 | 54 | 83986 | 41091 | 0.0006 | 0.0008 |
| TA Mlauli | Mwanza | 64 | 3 | 0.05 | 20 | 20 | 35569 | 17568 | 0.0006 | 0.0011 |
| TA Kanyenda | Nkhotabay_Kan | 122 | 11 | 0.09 | 81 | 107 | 116546 | 58425 | 0.0009 | 0.0014 |
| TA Malemia - Nsanje | Nsanje | 148 | 60 | 0.41 | 110 | 164 | 49281 | 23629 | 0.0033 | 0.0047 |
| TA Mbenje | Nsanje | 311 | 200 | 0.64 | 198 | 244 | 53559 | 25888 | 0.0046 | 0.0076 |
| TA Tengani | Nsanje | 148 | 60 | 0.41 | 125 | 175 | 41100 | 19840 | 0.0043 | 0.0063 |
| TA Goodson Ganya | Ntcheu | 92 | 26 | 0.28 | 155 | 201 | 143536 | 69259 | 0.0014 | 0.0022 |
| TA Malenga | Ntchisi | 99 | 3 | 0.03 | 11 | 15 | 50687 | 24788 | 0.0003 | 0.0004 |
| TA Chiwalo - Phalombe | Phalombe | 78 | 19 | 0.24 | 57 | 75 | 43933 | 21446 | 0.0017 | 0.0027 |
| TA Katumbi | Rumphi | 154 | 9 | 0.06 | 4 | 6 | 16028 | 7762 | 0.0004 | 0.0005 |

**S2 Table:** Traditional Authorities with available antigenaemia prevalence data and LF clinical cases data in Malawi.
