# Supplementary material for: The national distribution of lymphatic filariasis cases in Malawi using patient mapping and geostatistical modelling
Source: PLoS Negl Trop Dis. 2024 Mar 25;18(3):e0012056. doi: 10.1371/journal.pntd.0012056 (PMC11018277; doi:10.1371/journal.pntd.0012056)
Supplement: S1 File — (DOCX) [file pntd.0012056.s004.docx]

**S1 File.** Additional Methods and Results for Exploratory Analysis: Antigenaemia and Clinical Case Prevalence in Malawi.

## Methods: Exploratory Analysis: Antigenemia and Clinical Case Prevalence

The relationship between antigenemia prevalence and clinical case prevalence (cases per 100,000 population) at TA level was explored in areas where both data was available, i.e. clinical case mapping had been conducted and a village had been sampled in previous antigenemia study within TA area. A generalised linear model with the outcome variable as the number of clinical cases in each TA observation, assuming they followed a negative binomial distribution NB(μ, θ), was fitted with a single explanatory variable, antigenemia prevalence. Differences in TA population were accounted for in the model by including an logged offset term for population in each TA.

## Results: Relationship between antigenemia and clinical case prevalence


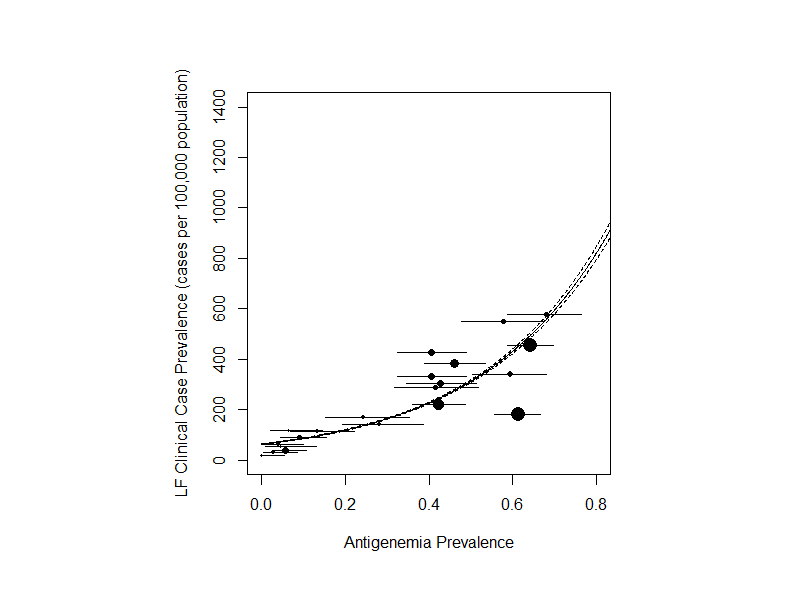
Exploratory analysis identified the relationship between antigenemia and clinical case prevalence in TAs where both observations were available. There was a strong positive relationship between antigenemia and clinical case prevalence (cases per 100,000 population), Supplementary Fig. TA areas with higher antigenemia prevalence had higher clinical case prevalence (Odds Ratios 24.9, 95% Confidence Intervals 23.3%-26.7%, p-value<0.001).

**Supplementary Fig 1:** The antigenemia prevalence for TA observations, weighted on the size of the point by the total number of people tested in that area, larger circle shows a greater number of individuals sampled, plotted against the clinical case prevalence (cases per 100,000 population).
