# Supplementary material for: The national distribution of lymphatic filariasis cases in Malawi using patient mapping and geostatistical modelling
Source: PLoS Negl Trop Dis. 2024 Mar 25;18(3):e0012056. doi: 10.1371/journal.pntd.0012056 (PMC11018277; doi:10.1371/journal.pntd.0012056)
Supplement: S2 File — (DOCX) [file pntd.0012056.s005.docx]

**S2 File:** Assessing goodness of fit of geostatistical model 1


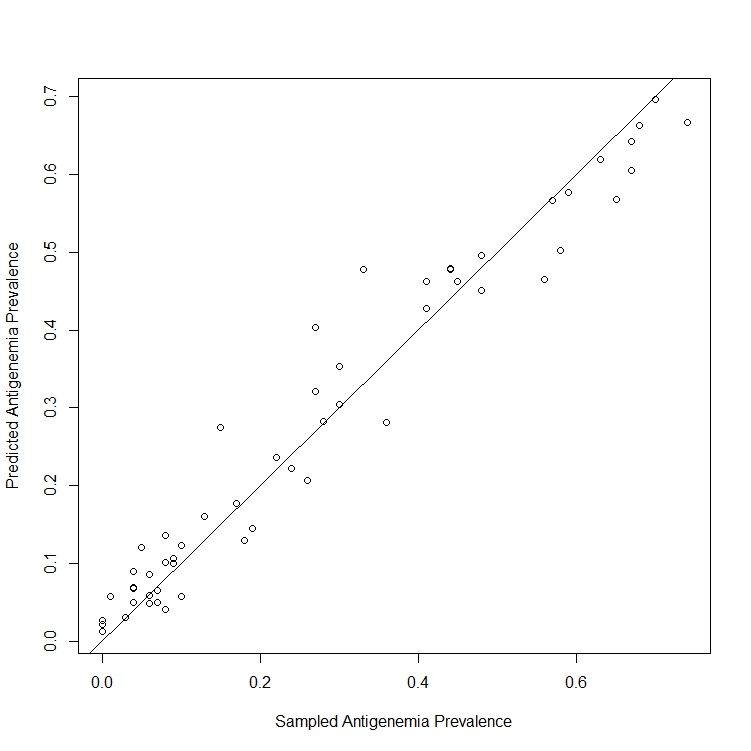
To assess the goodness of fit of the geostatistical model 1 predictions, the mean predicted antigenaemia prevalence was compared against sampled antigenaemia prevalence in Supplementary Fig 2.

**Supplementary Fig 2:** Predicted antigenaemia prevalence from geostatistical analysis step 1 compared against sampled antigeneamia prevalence.
